# Intraoperative Parathyroid Localization with Near-Infrared Fluorescence Imaging Using Indocyanine Green during Total Parathyroidectomy for Secondary Hyperparathyroidism

**DOI:** 10.1038/s41598-017-08347-6

**Published:** 2017-08-15

**Authors:** Le Cui, Yang Gao, Heping Yu, Min Li, Birong Wang, Tao Zhou, Qinggang Hu

**Affiliations:** 1 Department of Breast and Thyroid Surgery, Puai Hospital, Wuhan, 430030 China; 2 Department of Hepatobiliary Surgery, Union Hospital, Tongji Medical College, Huazhong University of Science and Technology, Wuhan, 430022 China; 3 State Key Laboratory of Luminescent Materials and Devices, South China University of Technology, Guangzhou, 510640 China; 4 Department of Otorhinolaryngology, Union Hospital, Tongji Medical College, Huazhong University of Science and Technology, Wuhan, 430022 China

## Abstract

The detection of all glands during total parathyroidectomy (TPTX) in secondary hyperparathyroidism (SHPT) patients is often difficult due to their variability in number and location. The objective of this study was to evaluate the feasibility of near-infrared fluorescence (NIRF) imaging using indocyanine green (ICG) for intraoperative parathyroid gland (PTG) localization in SHPT patients. Twenty-nine patients with SHPT were divided into two groups with or without intraoperative NIRF imaging. ICG was administered in patients undergoing intraoperative imaging, and the fluorescence of PTGs was assessed. Clinical and histopathologic variables were analyzed to determine factors associated with ICG uptake. Comparisons between NIRF and preoperative imaging, as well as differences between groups with or without NIRF imaging, were carried out to evaluate the efficacy of this technique. Most PTGs could be clearly identified, including one ectopic gland. The sensitivity of NIRF imaging is 91.1% in contrast to 81.82% for ultrasonography (US), 62.34% for ^99m^Tc-MIBI and 85.71% for computed tomography (CT). In addition, intraoperative NIRF imaging can reduce the operation time and improve the complete resection rate compared with the group not using it. Intraoperative NIRF imaging using ICG during TPTX is technically feasible and reliable for assisting surgeons in detecting and confirming PTGs.

## Introduction

Secondary hyperparathyroidism (SHPT), a common serious and progressive complication associated with chronic kidney disease (CKD), is characterized by persistently elevated serum parathyroid hormone (PTH), parathyroid gland (PTG) hyperplasia and mineral metabolism abnormalities^1^. SHPT patients present with various bone disorders and cardiovascular disease, leading to substantial morbidity or mortality^2^. Total parathyroidectomy (TPTX) is an effective approach for severe SHPT patients who are resistant to pharmacological treatments^3^. Removal of all PTGs is the essence of successful PTX, which prevents continuous residual gland stimulation in the CKD environment. However, it is difficult to resect all PTGs because of their variability in number and location, causing enormous challenges to parathyroid identification for even experienced surgeons^4^. The persistent and recurrent rate ranges between 10% and 30% due to incomplete resection^5–7^. Nevertheless, this situation could be easily improved with an effective parathyroid identification method. Although there have been advances in nuclear, ultrasound (US) and computed tomography (CT) imaging, the sensitivity of ^99m^Tc-MIBI is reported to range between 50% and 80%, that of US ranges between 40% and 85%, and that of CT ranges between 60% and 80%^8–14^. Worse still, these preoperative images are difficult to translate into intraoperative real-time anatomical information to help surgeons make an immediate decision. Thus, there is an urgent need for a safe, convenient and sensitive real-time intraoperative approach for PTG identification.

Near-infrared fluorescence (NIRF) imaging has gained increasing attention in recent years for image-guided surgery due to their strong anti-interferences of optical absorption, high tissue penetration, low photo-damage and minimal auto-fluorescence from tissue^15^. Therefore, NIR methylene blue (MB) and blue aminolevulinic acid (ALA) were used to identify the normal PTG or parathyroid adenoma in several literature reports^4, 16, 17^. However, the toxicity and lack of data demonstrating the improvement of outcomes have restricted its popularization in parathyroid surgery. Thus, a desired NIR probe is the critical element in image-guided parathyroid surgical procedures. Indocyanine green (ICG) is an anionic, water-soluble tricarbocyanine molecule that could emit strong fluorescence with a peak wavelength approximately 830 nm once excited by NIR light when it binds to proteins^18^. Because of much better tissue penetration and biocompatibility, ICG has been applied to general surgical and oncologic procedures, including intraoperative cholangiography, the assessment of anastomotic perfusion and sentinel node mapping for decades^19–22^. In a pre-clinical trial, the location of PTGs could be visualized using ICG through a NIRF imaging technique in dog models^23^. Subsequently, Berber *et al*.^24^ further assessed the usefulness of ICG fluorescence imaging in patients undergoing PTX procedures for primary hyperparathyroidism, demonstrating that ICG could reliably localize the PTGs with a high detection rate of 92.9% of 112 glands in 33 patients. Additionally, a recent study evaluated the vascularization of PTG remnants by ICG fluorescence imaging during subtotal PTX to predict postoperative remnant function^25^. However, no study has yet reported its application for intraoperative parathyroid localization in patients with SHPT. Herein, we investigated the real-time intraoperative ICG fluorescence imaging of hyperplasic PTGs during TPTX for SHPT and prospectively assessed the feasibility, utility and safety of image-guided surgery for TPTX.

## Results

### Patients

**Figure 1:**
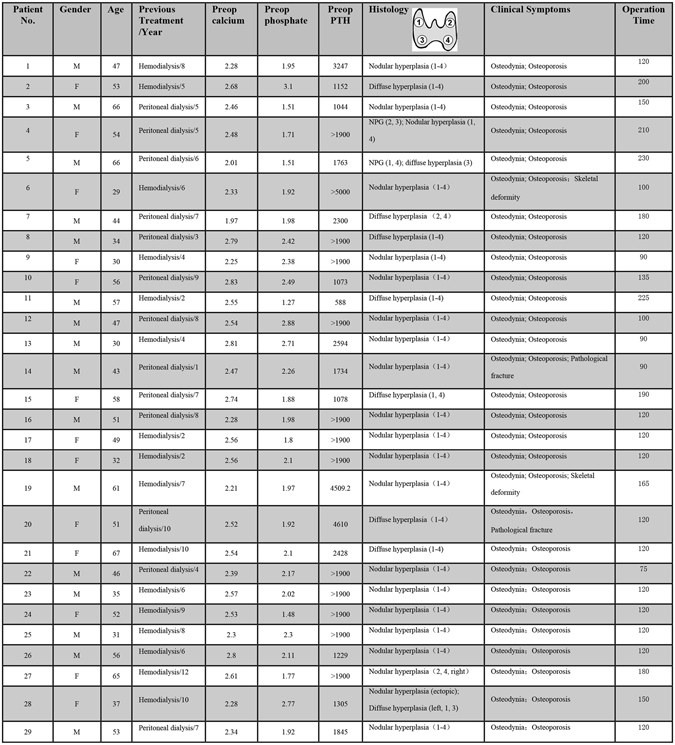
Patient, histopathologic and surgical characteristics. Abbreviations: M, Male; F, Female; Preop, preoperative; PTH, Parathyroid hormone; NPG, Normal parathyroid gland.

Twenty-nine patients planning TPTX were entered in this study. These patients were divided into two groups. The TPTX group without ICG fluorescence imaging included 9 patients (No. 1 to No. 9), from whom 42 lesions were resected. Of these 42 lesions, 33 lesions (78.57%) were confirmed to be PTGs. Another group undergoing TPTX with ICG fluorescence imaging included 20 patients (No. 10 to No. 29), from whom 82 lesions were resected. Of these 82 lesions, 77 lesions (93.90%) were confirmed to be PTGs. Thus, the resection rate in the group with ICG fluorescence imaging was significantly higher than that in the group without ICG fluorescence imaging. Additionally, there were no statistically significant differences between the two groups regarding demographic and preoperative clinical data. The patients presented with various clinical symptoms, but the main complaints were osteodynia, osteoporosis, skeletal deformity and pathological fractures. Details about patient characteristics, histology results, and operation times are shown in Figure 1. We did not encounter in-hospital mortality in either series, and postoperative surgery-related complications such as bleeding or nerve injury did not occur in either group. Still, we had four patients (2 cases in each group) with persistent hyperparathyroidism after surgery, a fact that we interpreted as missing glands.

### Intraoperative NIR fluorescence imaging

**Figure 2:**
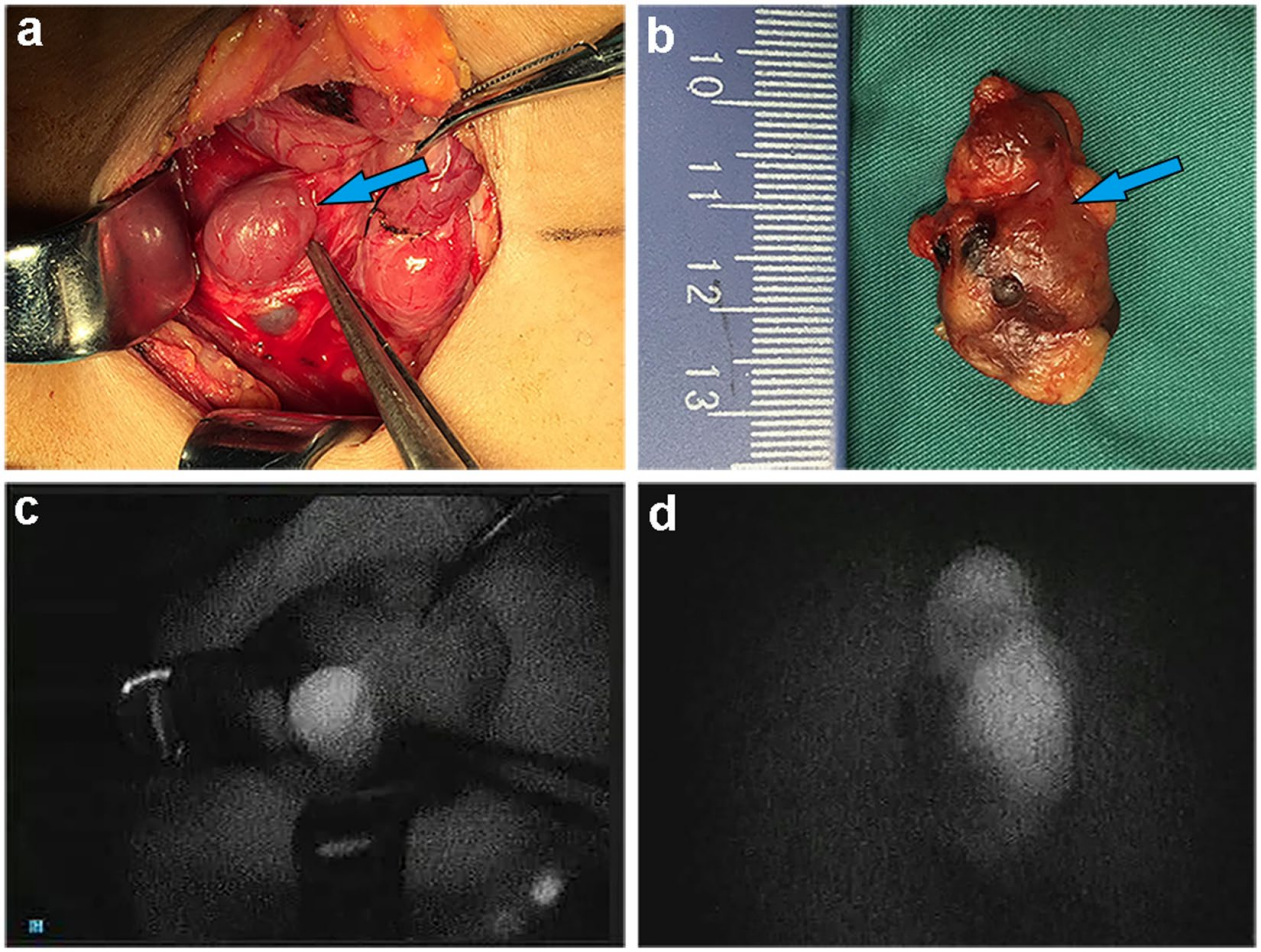
Representative hyperplastic PTG and intraoperative NIRF image using ICG. (**a**,**b**) Bright-field and (**c**,**d**) fluorescent images of hyperplastic gland intraoperative and after resection.

**Figure 3:**
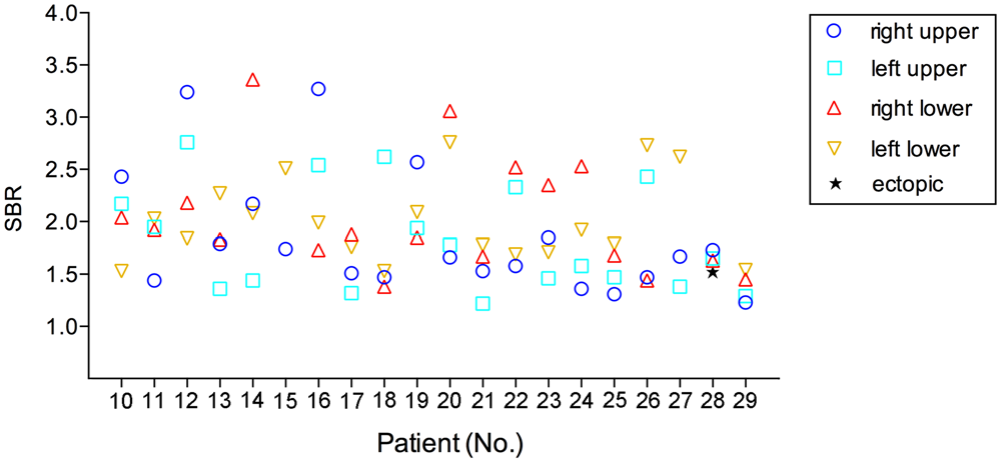
The SBR distribution of total 77 resected glands of 20 patients with intraoperative NIRF image using ICG.

**Figure 4:**
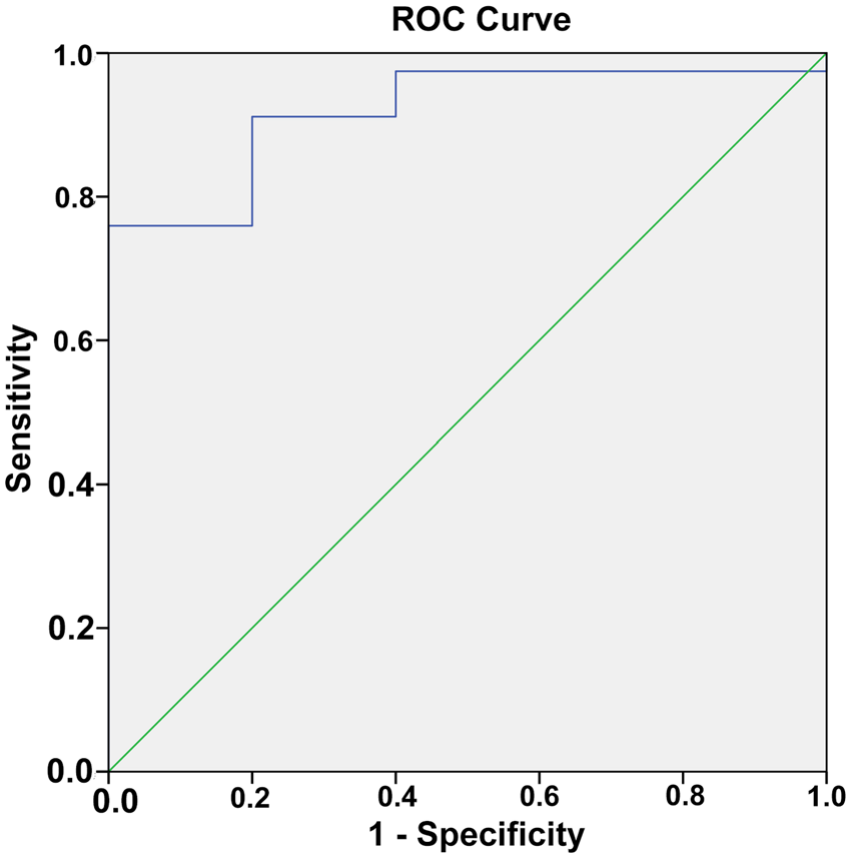
The ROC curve of NIRF image using ICG based on the SBRs.

**Figure 5:**
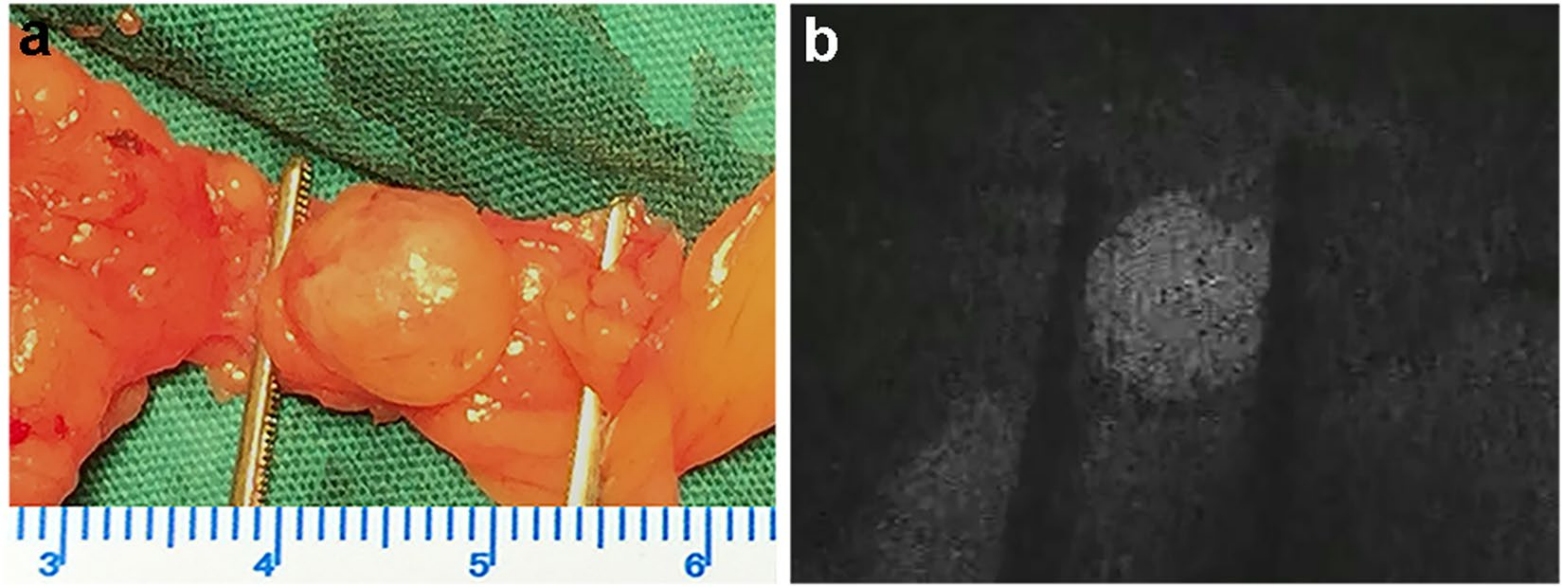
Bright-field (**a**) and fluorescent image (**b**) of the ectopic PTG in thymus.

Seventy-seven of the resected glands of the group with ICG fluorescence imaging were hyperplasia according to the postoperative pathology. Intraoperative ICG fluorescence imaging successfully identified most pathologic enlarged PTGs. Figure 2 shows a representative example of the intraoperative fluorescence signal in a PTG. The fluorescence intensity of the PTG was greater than that of all other tissues. The thyroid exhibited a weaker fluorescence signal than the parathyroid but was consistently stronger than that of muscle, fat, and other surrounding tissues, which showed almost no fluorescence. The average SBR of the identified PTGs was 1.92 ± 0.52. Figure 3 shows the SBR of all resected glands from the total 20 patients. The ROC curve of NIRF was also made based on SBR, including 5 false-positive and 2 false-negative cases (Fig. 4). The mean time interval between the administration of ICG and detection of the PTGs was 75 ± 35 minutes. The PTGs could be identified up to 225 minutes after administration, providing sufficient imaging time until resection in these patients. Notably, 1 patient (No. 28) had an ectopic PTG in the thymus, and the fluorescence imaging also successfully identified it with an SBR = 1.55 (Fig. 5), demonstrating that ICG can also be taken up even in ectopic PTG.

### Factors associated with SBR

To analyze the clinical variables associated with the SBR of PTGs, analysis was performed to determine factors associated with ICG uptake. As shown in Table 1, by Pearson’s Chi-squared test or Fisher’s exact test analysis, the age, gender, dialysis time, calcitriol pulse therapy, preoperative phosphate and calcium, and histology showed no correlation with the SBR. A significantly higher degree of fluorescence was seen in patients presenting with a preoperative PTH level >1900 pg/mL (*P* < 0.05) and in PTGs larger than 10 mm (*P* < 0.01). There was also a tendency toward increased SBR in patients with hemodialysis (*P* < 0.01).

**Table 1:** Analysis of parathyroid fluorescence by clinical and histopathologic parameters.

Parameter	Number	SBR (mean)	*P* value
Age			
<50 year	9/20	1.94	0.38
>50 year	11/20	1.91	
Gender			
Male	11/20	1.98	0.26
Female	9/20	1.87	
Dialysis modality			
Hemodialysis	12/20	1.78	<0.01
Peritoneal dialysis	8/20	2.13	
Dialysis time			
<5 year	6/20	1.93	0.61
>5 year	14/20	1.93	
Calcitriol pulse therapy			
Yes	12/20	1.92	0.53
No	8/20	1.95	
Preoperative calcium			
<2.75 mmol/L	17/20	1.93	0.71
>2.75 mmol/L	3/20	1.96	
Preoperative phosphate			
<1.62 mmol/L	2/20	1.84	0.67
>1.62 mmol/L	18/20	1.94	
Preoperative PTH			
<1900 pg/mL	7/20	1.88	<0.05
>1900 pg/mL	13/20	2.07	
Parathyroid size (max diameter)			
≤10 mm	16/77	1.47	<0.01
>10 mm	61/77	2.04	
Gland histology			
Diffuse hyperplasia	17/77	1.87	0.69
Nodular hyperplasia	60/77	1.93	

### Comparison of NIRF imaging with preoperative imaging

**Figure 6:**
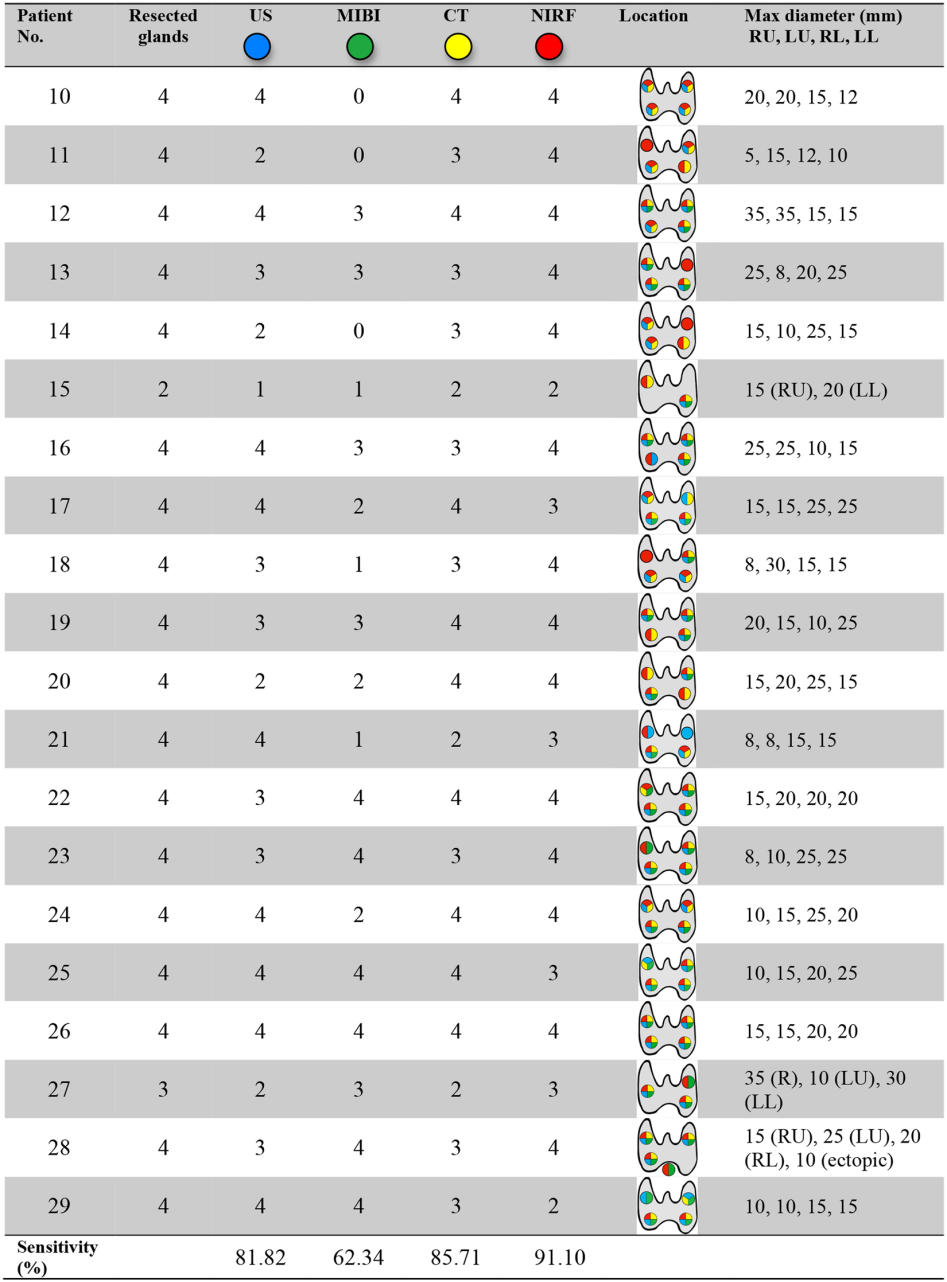
Number and location of the detected glands by different modalities and the max diameter of resected glands. Abbreviations: R, right; L, left; RU, right upper; LU, left upper; RL, right lower; LL, left lower.

Patients in the ICG fluorescence imaging group all underwent preoperative imaging (US, CT, ^99m^Tc-MIBI) and intraoperative NIRF imaging. We recorded the number and location of PTGs detected by each imaging modality. Figure 6 shows characteristics of the four imaging modalities. Preoperative US successfully detected 63 PTGs in 20 patients, and the sensitivity of US was 81.82%. There were 48 PTGs in 17 patients identified by ^99m^Tc-MIBI, and the sensitivity was 62.34%. The preoperative CT identified 66 PTGs in 20 patients, and the sensitivity was 85.71%. The receiver operating characteristic (ROC) curve was used to analyze the efficacy of NIRF imaging, with SBR being regarded as the test variable. The NIRF shows high accuracy with the area under curve (AUC) of 0.919 as shown in Fig. 4. Determination of the minimum SBR threshold can define whether a PTG will fluoresce and calculate the sensitivity of NIRF imaging. Based on our experience and the ROC curve, the PTGs can be clearly and quickly identified by the NIRF imaging during surgery with a sensitivity of 91.1% and a specificity of 80% when the threshold is defined as 1.35.

### Comparison between the two groups

**Figure 7:**
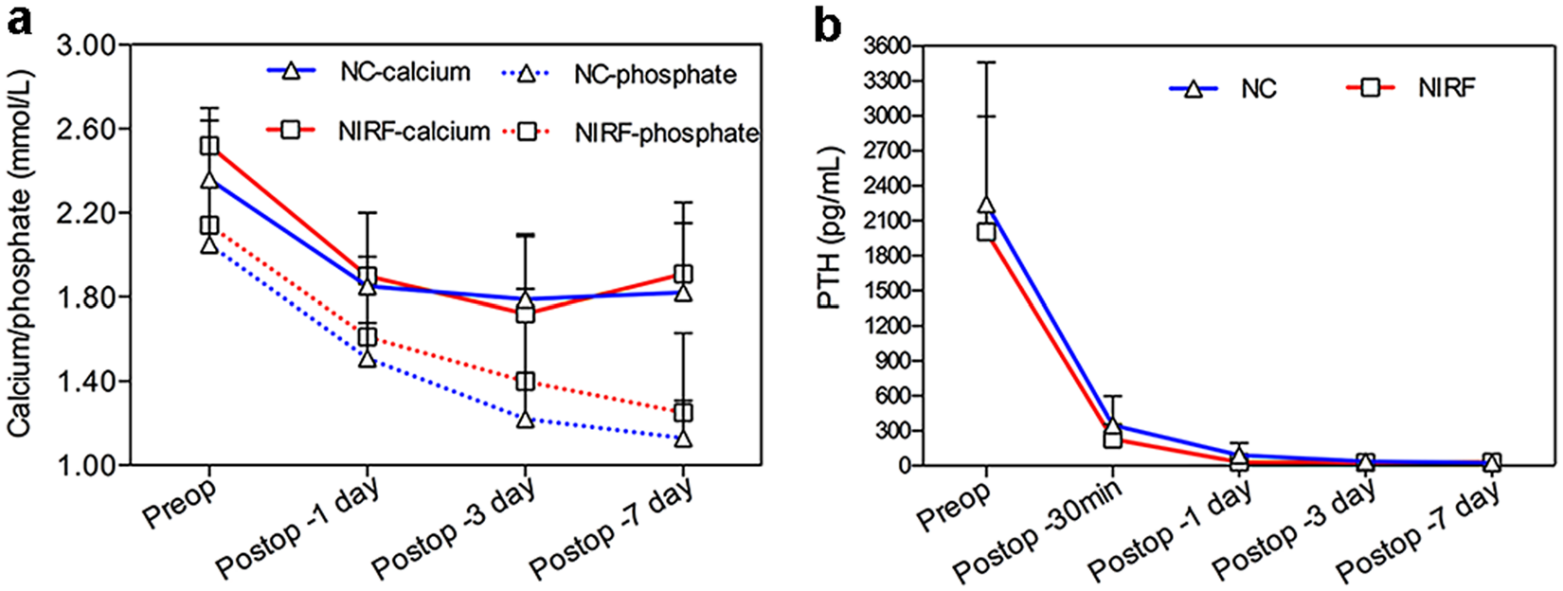
(**a**) The variations of preoperative and the 1^st^, 3^rd^, 7^th^ days postoperative serum calcium and phosphorus. (**b**) The variations of preoperative and the 1^st^, 3^rd^, 7^th^ days postoperative PTH. Negative control (NC): patients without intraoperative NIRF imaging using ICG.

To demonstrate the application value of intraoperative imaging, we compared the differences between the group with or without ICG fluorescence imaging. As shown in Fig. 7, there was no significant difference between the two groups preoperatively and postoperatively regarding serum calcium, phosphorus and PTH variations (*P* > 0.05). The symptomatic relief rate was 100%, and no complication occurred. These results demonstrated that the two groups could achieve the same therapeutic effect in patients with complete resection. Unfortunately, each group had 2 cases with persistent hyperparathyroidism after surgery that we interpreted as missing glands. The incidence rates were 10.0% (2/20) vs 22.2% (2/9), which represented a significant difference between the two groups (*P* < 0.05), demonstrating that the high sensitivity of intraoperative ICG fluorescence imaging could help surgeons to identify PTGs for complete resection. Moreover, the mean operation time of the group with or without ICG fluorescence imaging was 130 min vs 156 min (*P* < 0.05), respectively, demonstrating intraoperative ICG fluorescence imaging could reduce the operation time.

## Discussion

Although PTX is a commonly performed and overall successful procedure, intraoperative detection of the gland is still challenging. Surgical failure is often due to missing resection, especially for ectopic glands^26^. Recently, many attempts have been made to aid intraoperative localization of PTGs. Among these efforts, NIRF imaging of PTGs using an exogenous fluorophore provides a promising approach to overcome this dilemma. Several studies employed MB as the fluorophore for parathyroid adenoma imaging. However, no study explored the feasibility for pathological enlarged parathyroid^16, 17^. Worse still, recent data have suggested that MB is associated with toxic metabolic encephalopathy, particularly in those patients taking serotonin reuptake inhibitors^27, 28^. The use of photosensitizers such as ALA is another emerging technology. However, this technology can be beneficial to only 50% of patients for detection the glands during the operation^4^. In addition, the photosensitizer-related complications are still a limitation, preventing its further application^29^. NIR-IR auto-fluorescence imaging is also a method for intraoperative real-time localization of PTGs. Although satisfactory performance of auto-fluorescence has been reported recently^30, 31^, the equipment for this method is not yet commercially available, and this technique has not been verified in SHPT patients so far.

An ideal fluorophore to facilitate intraoperative localization should be safe and convenient^32^. ICG, a NIR probe that has been approved by the FDA for decades clinically, was reported to be associated with less than 0.05% of serious adverse events^33^. What’s more, it only needs intravenous injection before surgery without extra work pre- or post-operatively. More importantly, ICG has a property of accumulation in pathological PTGs, which attributes to the higher fluorescence intensity than the surrounding tissues. These advantages make ICG an ideal NIR probes for hyperplastic PTGs localization.

Presently, the mechanisms that mediate the preferential accumulation of ICG in hyperplastic glands relative to other normal tissue remain unknown. It is known that hyperplastic PTGs have high metabolic activity because the parenchymal cells contain a proportionally higher number of mitochondria than other tissues^34^. In addition, hyperplastic PTGs always have architectural destruction due to disorder arrangement of the hyperplastic cells^35^. The proportion of hyperplastic cells increases in the parathyroid gland significantly. The types of cells tend to be consistent, with single main cells, clear cells or acidophilic cells occupying the whole gland. There is a significant increase in fiber septums whereas a decrease in adipose tissues. In addition, old hemorrhage and calcification always occur in hyperplastic gland^36^. Thus, we hypothesized that the preferential accumulation of ICG and subsequent fluorescence in PTGs may be related to the high metabolic activity and structural disorder of hyperplastic PTGs. When ICG flows through the PTGs, it will be taken up more by the hyper-functional cells. Other tissue cells (e.g., fat, muscle, lymph node, and other surrounding neck tissues) can rapidly clear out their ICG and appear dark, while ICG has delayed excretion due to architectural destruction in hyperplastic PTGs. According to the data we have now obtained (Table 1), the SBR is higher in patients with large PTGs and high PTH levels, which supports our hypothesis. This is only a preliminary exploration. Further studies, such as molecular and immunohistochemical research, are needed to understand the mechanism and pattern of ICG accumulation in PTGs.

A comparison was made between lesions detected by NIRF imaging and traditional preoperative imaging modalities. US and CT belong to morphological imaging, and the sensitivity of detection depends on the size of the lesion and density difference contrast to adjacent tissue. The sensitivity values of US and CT in our study were 81.82% and 85.71%, respectively, indicating a unsatisfactory performance in identifying the PTGs. ^99m^Tc-MIBI is one method of radionuclide imaging, which has a higher sensitivity to hyper-functional PTGs than normal glands. The potential role of ^99m^Tc-MIBI in the preoperative localization of PTGs in SHPT patients has been investigated by several studies^10, 37^. The sensitivity of ^99m^Tc-MIBI in our study was 62.34%, which was similar to that in recent reports of the variable accuracy of planar parathyroid scintigraphy using ^99m^Tc-MIBI in SHPT ranging from 35% to 90%^38, 39^. However, the sensitivity of these preoperative imaging was lower than that of intraoperative ICG fluorescence imaging that had a 91.1% sensitivity according to our data. Furthermore, ICG fluorescence imaging can transfer the location into real-time information for accurate and rapid surgery.

The complete resection rate of ICG fluorescence imaging group was remarkably higher, whilst the operation time and persistent hyperparathyroidism rate were less than these in the group without using it. On the other hand, variations in postoperative calcium, phosphorus and PTH, as well as complications and the relief rate, showed no difference between the two groups. These results suggest that NIRF imaging can be completed successfully for most patients and may be associated with a better outcome and reduced operating time with no difference in the therapeutic effect. Notably, there were also 2 cases in the ICG group with persistent hyperparathyroidism after surgery that we interpreted as missed resection. Thus, several glands presented as false negative upon NIRF imaging, indicating that fluorescence imaging combined with careful visual inspection is still necessary for these patients.

Although we have made some encouraging progress in the research of intraoperative NIRF parathyroid imaging, an invincible drawback of this fluorescence technique is given by the optical property of light-penetrating tissue. NIR is generally known to have the deepest penetration among the spectrum, which can reach a tissue depth of only 10 mm^40^. Thus, surgery with broad exposure and careful dissection of the area close to the glands is still necessary. Another potential pitfall using this technique is the background signal in the thyroid gland that may interfere with the identification of PTG, and this situation may worsen with normal PTGs, which often have lower SBRs than the hyperplastic ones. Thus, the interval between ICG injection and imaging should be longer to obtain a good SBR. Further studies are required to determine the optimal dosage of ICG and interval time between administration and imaging. Another cause of this situation is the poor specificity of ICG to parathyroid glands. Unlike antibody binding to an antigen showing the specificity, ICG could also accumulate in other tissues due to the lack of active targeting, resulting in a high background signal and a low SBR. Differences in contrast are mainly due to differences in retention of the dye within tissues and variability in clearance. Active targeting groups, such as antibodies coupling ICG, will be a good strategy to settle this problem. The conjugation of ICG with antibodies that combines the optical properties of ICG with the specific recognition ability of the antibodies to the antigens on the surface of tumor cells has made some progress. For instance, some proteins such as integrin, VEGF, and HER-2, are overexpressed on the surface of tumor cells compared with normal cells. Many studies have utilized these antibodies to conjugate with ICG and have achieved excellent effects for tumor imaging^41–43^. These studies may provide an approach to solve the specificity of ICG to the parathyroid gland in the future. Currently, ICG fluorescence imaging might be not a complete substitute for careful visual inspection during operation, but this technique can be used as an auxiliary to help localize hyperplastic PTGs, which may have more sensitivity than preoperative images and provide real-time information to surgeons.

## Conclusion

In this study, ICG was investigated for intraoperative NIRF imaging of PTGs in patients with SPTH during TPTX for parathyroid localization. The results demonstrated that this technique could easily localize the parathyroid and possess a high resection rate of 93.9%. The fluorescent intensity of PTGs was stronger than that of the surrounding tissues, with an average SBR of 1.92 ± 0.52. Analysis revealed that the SBR of PTGs was associated with the dialysis modality, preoperative PTH level and size of PTGs. The sensitivity of intraoperative ICG fluorescence imaging was higher than that all of preoperative imaging modalities. Furthermore, surgeons can fast and easily identify the PTGs when using the intraoperative fluorescence imaging, as well as ensure the therapeutic efficacy. Thus, intraoperative ICG fluorescence imaging has promising application prospects in real-time PTG localization. Utilizing this technique has the potential to help complete resection and improve the clinical outcome for SHPT patients.

## Subjects and Methods

### Patients

Twenty-nine patients undergoing TPTX from SHPT caused by CKD at the Department of Thyroid and Breast Surgery of Puai Hospital were recruited and participated in the study. Our inclusion criteria based on the kidney disease outcomes quality initiative (KDOQI) guideline for TPTX were as follows: resistance to available medical therapy; PTH levels greater than 800 pg/mL with hyper-calcemia and/or hyper-phosphatemia; severe clinical symptoms such as osteodynia, pruritus, pathological fractures, metastatic calcifications, and calciphylaxis; maximum diameter of PTG > 1 cm. Patients with an ICG allergy were excluded. This study was approved by the Medical Ethics Committee of Tongji Medical College and was performed according to the ethical standards of the Helsinki Declaration of 2013. Written informed consent was obtained from all individuals before surgery. Patients were divided into 2 groups with or without ICG fluorescence imaging. All procedures were performed by the same surgeon. Standardized care diagnostic work-up that, for our center, included preoperative cervical US, ^99m^Tc-MIBI and CT for preoperative planning, were performed in all patients. The number and location of PTGs detected by each method were recorded.

### Fluorescence technique and intraoperative imaging

The fluorescent imaging system was provided by AISERY Co. (Beijing, China). Briefly, light-emitting diodes with an excitation wavelength of 760 nm as the light source were aligned in the outer periphery of the detector, in which a charge-coupled device (CCD) camera is in the center. The detector is positioned above the surgical field when the fluorescent image is captured. An optical filter in front of the CCD camera can filter out emission light with a wavelength below 830 nm, and the fluorescence signal is then transmitted to the computer *via* an image capture card. Finally, the fluorescent image is created and presented on the display screen.

ICG (0.5 mg/kg) was intravenously administered 1 h before the start of anesthesia. A four-gland exploration was performed for patients undergoing TPTX. After sub-platysmal dissection, retraction, and initial exploration, visual inspection combined with NIRF imaging was used for the identification of PTGs. To investigate the fluorescence signal, images were captured by the detector after full exposure. All suspected lesions were resected during surgery, and histologic analysis of frozen sections was conducted. The resected lesions were imaged again to identify the fluorescence signals. Postoperative histopathologic assessment of the resected lesions served as the gold standard for parathyroid identification. In addition, confirmation of surgical success was attained by a significant decrease (50%) of the intraoperative PTH from the baseline serum concentration 20 minutes after removal of the glands^3, 44^.

### Assessment of ICG fluorescence imaging

The fluorescence intensity (*I*) of the PTGs and regions of interest were measured using Image J software. Performance metrics of ICG was assessed by the signal-to-background ratio (SBR) compared with the surrounding peripheral tissue (adipose tissue or thyroid) of the parathyroid. The SBR was calculated as follows: SBR = (*I*_*PTG*_ − *I*_*noise*_)/(*I*_*peripheral tissue*_ − *I*_*noise*_). The noise signal was recorded in a region outside the surgical field.

The clinical and histopathologic variables studied included age, gender, dialysis modality and time, preoperative calcium, preoperative phosphorus, PTH, calcitriol pulse therapy, parathyroid size and parathyroid histopathology. Analysis was conducted to determine the factors associated with ICG uptake. The sensitivity of four modalities (US, CT, ^99m^Tc-MIBI and NIRF) was also evaluated. To evaluate the efficacy of NIRF in detection of the PTGs and to establish the optimal cut-off point, the receiver operating characteristic (ROC) curve was made. To compare the difference between groups with and without ICG fluorescence imaging, the serum levels of calcium, phosphorus and PTH were regularly assessed at the 1^st^, 3^rd^, and 7^th^ days after the operation, respectively. In addition, the operation time, complication, recurrence and persistence rate and symptomatic relief rate were also compared.

### Statistical analysis

Statistical analysis was performed using SPSS software (Version 20.0; Chicago, IL). To compare the characteristics between different groups, an independent-sample *t* test was used. All categorical variables were analyzed statistically using Pearson’s Chi-squared test or Fisher’s exact test. The ROC curve of NIRF was made by SPSS. *P* < 0.05 was considered significant.
